# The Bark of the Spruce *Picea jezoensis* Is a Rich Source of Stilbenes

**DOI:** 10.3390/metabo11110714

**Published:** 2021-10-20

**Authors:** Andrey R. Suprun, Alexandra S. Dubrovina, Olga A. Aleynova, Konstantin V. Kiselev

**Affiliations:** Laboratory of Biotechnology, Federal Scientific Center of the East Asia Terrestrial Biodiversity, Far Eastern Branch of the Russian Academy of Sciences (FEB RAS), 690022 Vladivostok, Russia; suprun.hi@gmail.com (A.R.S.); dubrovina@biosoil.ru (A.S.D.); aleynova@biosoil.ru (O.A.A.)

**Keywords:** astringin, bark, isorhapontin, *Picea jezoensis*, piceatannol, piceid, resveratrol, stilbenes

## Abstract

Stilbenes are plant phenolic secondary metabolites that show beneficial effects on human health and possess high antifungal activity against plant pathogens. Currently, a search for plant sources with high stilbene levels is relevant, since stilbene content in various plant species can vary substantially and is often at a low level. In this paper, the bark and wood of *Picea jezoensis* were analyzed for the content and composition of stilbenes and compared with other known stilbene sources. The HPLC-MS analysis of *P. jezoensis* bark and wood extracted with different solvents and at different temperatures revealed the presence of 11 and 5 stilbenes, respectively. The highest number of stilbenes of 171 and 229 mg per g of the dry weight (mg/g DW) was extracted from the bark of *P. jezoensis* using methanol or ethanol at 60 °C for 2 h. *Trans*-astringin, *trans*-piceid, and *trans*-isorhapontin prevailed over other stilbenoids (99% of all detected stilbenes). The most abundant stilbene was *trans*-isorhapontin, reaching 217 mg/g DW or 87% of all stilbenes. An increase in the extraction time from 2 to 6 h did not considerably increase the detected level of stilbenes, while lower extraction temperatures (20 and 40 °C) significantly lowered stilbene yield. The content of stilbenes in the *P. jezoensis* bark considerably exceeded stilbene levels in other stilbene-producing plant species. The present data revealed that the bark of *P. jezoensis* is a rich source of stilbenes (primarily *trans*-isorhapontin) and provided effective stilbene extraction procedures.

## 1. Introduction

Plant secondary metabolites possess valuable biologically active properties and are applied directly as drugs or as raw materials for further modifications [1,2]. The most valuable metabolites are actively used in modern medicine and have significant applications in the food and cosmetic industries. Studying the composition and content of secondary metabolites in the plant material promotes the discovery of new rich sources of valuable biologically active compounds and the development of new effective metabolite production methods [2,3]. The spectrum of plant secondary metabolites includes tens of thousands of substances that are divided into several major classes, including alkaloids (mainly heterocyclic compounds), terpenoids or isoprenoids, natural phenols, and several thousands of other minor compounds, e.g., some non-protein amino acids, amines, betalains, allicins, cyanolipids, etc. [1,2].

Stilbenes are natural compounds occurring in a number of unrelated plant families, including Vitaceae (grape), Fabaceae (peanut), or Pinaceae (pine). Stilbenes, with the most-studied compound resveratrol (3,5,4′-trihydroxy-*trans*-stilbene), occupy a special place among the secondary metabolites of plants due to their well-known beneficial biological activities, including antioxidant, anticancer, antiviral, anti-inflammatory, anti-microbial, cardio-protective, neuroprotective, phytoestrogenic, and radioprotective properties [4,5,6,7,8].

Stilbene structures are based on the structure of pinosylvin (3,5-dihydroxy-*trans*-stilbene), resveratrol (3,5,4′-trihydro’xy-*trans*-stilbene), and piceatannol (3,4,3′,5′-tetrahydroxy-*trans*-stilbene), which are the key precursors in the stilbene biosynthesis [9]. The monomeric stilbenes may be metabolized to form other stilbenes, such as pterostilbene or isorhapontigenin via methylation by an O-methyltransferase [9,10], astringin or piceid via glycosylation by glucosyltransferases [9,11], or viniferins via oxidation by polyphenol oxidase (PPO) [12,13,14,15].

Stilbenes are inducible compounds in grapes and many other plant families: while stilbenes are normally detected in trace amounts, their content sharply increases by 2–30 times 12–24 h after induction with ultraviolet, salicylic acid, or methyl jasmonate [16,17,18,19,20,21,22]. However, this increase is transient, i.e., 24–48 h after the stimulating effect, the content of stilbenes again returns to trace amounts. In contrast to Vitaceae species, spruce and other Pinaceae plants constitutively accumulate stilbenes [23], while the content of stilbenes in spruce or pine is weakly induced, and it does not fall to trace amounts 24–48 h after a treatment [24,25,26]. Therefore, Pinaceae species can represent a valuable stable source of stilbenes. Pinaceae species mainly produce such stilbenes as *trans*-astringin, *trans*-piceid, and *trans*-isorhapontin [6], and their content varied depending on the used tissues, season, and plant age [25]. A search for rich sources of stilbenes is an important task in modern plant biotechnology.

The highest content of stilbenes among analyzed plant families was reported in the bark of Pinaceae plants, including the bark of Norway spruce *Picea abies* and Black pine *Pinus nigra* with the stilbene content reaching 58–60 mg/g DW [27,28]. To the best of our knowledge, other spruce species besides *Picea abies* were not analyzed for stilbene contents in the bark and wood. The aim of this study was to characterize stilbene content and repertoire in the bark of spruce *Picea jezoensis* and compare it with that in the wood and needles of *P. jezoensis.* We also aimed to find the most efficient conditions for the stilbene extraction procedure from the bark of *P. jezoensis*. Thus, this paper firstly reported the content and composition of stilbenes in the bark of *P. jezoensis*. Using different extraction procedures, we were able to isolate up to 251 mg/g DW of total stilbenes from the bark, which considerably exceeds stilbene levels reported for other plant materials.

## 2. Results

### 2.1. Stilbene Identification and Quantification in the Bark and Wood of P. jezoensis by HPLC-MS-UV

When processing spruce wood for logging, the main final products include lumber and, in addition, a lot of by-products that are not included into the main production: small branches, needles, and bark. Thus, we decided to analyze the content of stilbenes in the bark, needles, and wood of *P. jezoensis* using HPLC-MS-UV after ethanol extraction, which is a standard extraction procedure for stilbenes from the plant material. The results obtained showed that the spruce bark contained considerably higher levels of stilbenes (165 mg/g DW) than the needles and wood of *P. jezoensis* (Table 1). In turn, the needles of *P. jezoensis* contained considerably higher levels of stilbenes (8 mg/g DW) than the wood of *P. jezoensis*. Stilbenes were present only in trace amounts in the wood of *P. jezoensis* (only 0.07 mg/g DW, Table 1).

**Figure 1:**
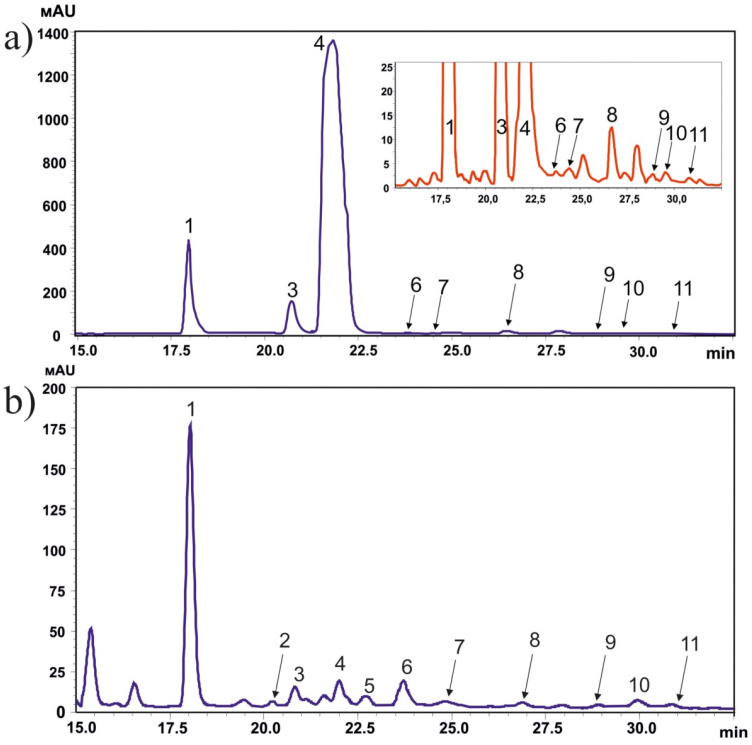
Comparison of HPLC-UV chromatographic profiles for the methanolic extracts of spruce bark (**a**) and needles (**b**) recorded at 310 nm. Spruce bark and needles were collected in winter. *Trans*-astringin (1, retention time 18.0 min), *cis*-astringin (2, 20.2 min), *trans*-piceid (3, 20.8 min), *trans*-isorhapontin (4, 21.9 min), *trans*-piceatanol (5, 22.6 min), *cis*-piceid (6, 23.9 min), *cis*-isorhapontin (7, 24.5 min), *trans*-resveratrol (8, 26.5 min), *trans*-isorhapontigenin (9, 28.8 min), *cis*-resveratrol (10, 29.7 min), *cis*-isorhapontigenin (11, 20.2 min).

**Figure 2:**
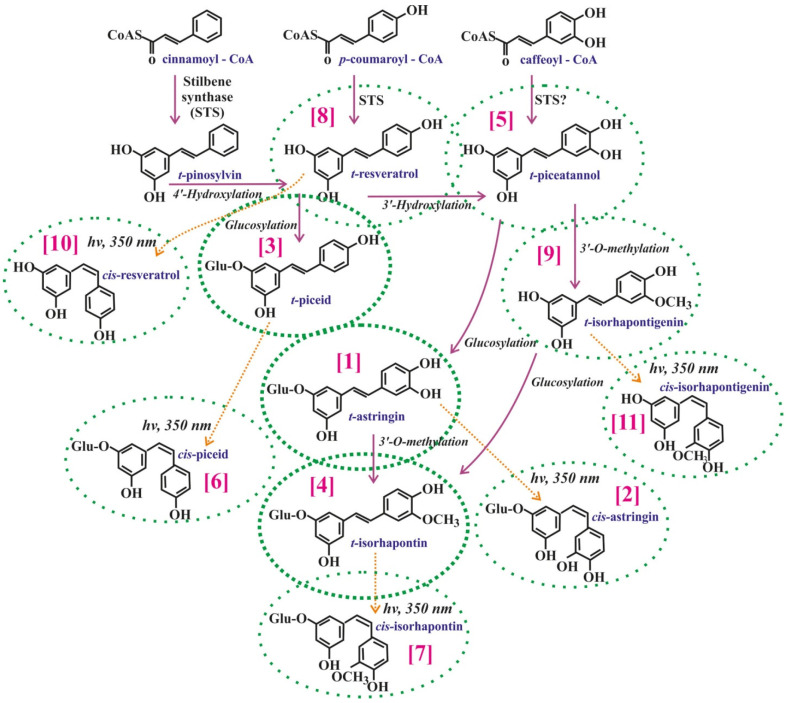
Hypothetical biosynthetic pathway for stilbene formation in Pinaceae [25]. Circles with dotted lines depict 11 stilbenes detected in the needles of spruce *Picea jezoensis*: *trans*- and *cis*-astringin [1,2], *trans*- and *cis*-piceid [3,6], *trans*- and *cis*-isorhapontin [4,7], *trans*-piceatannol [5], *trans*- and *cis*-resveratrol [8,10], and *trans*- and *cis*-isorhapontigenin [9,11]. *Trans*-astringin [1], *trans*-piceid [3], and *trans*-isorhapontin [4] marked with thick dotted lines because the content of these compounds is much higher than the other stilbenes. The peak numbers for detected stilbenes in square brackets are shown as in Figure 1.

The HPLC-MS-UV analysis showed the presence of 11 stilbenes in the *P. jezoensis* ethanol extracts (Figure 1 and Figure 2). The stilbenes included *trans*-astringin (retention time 18.0 min), *cis*-astringin (20.2 min), *trans*-piceid (20.8 min), *trans*-isorhapontin (21.9 min), *trans*-piceatanol (22.6 min), *cis*-piceid (23.9 min), *cis*-isorhapontin (24.5 min), *trans*-resveratrol (26.5 min), *trans*-isorhapontigenin (28.8 min), *cis*-resveratrol (29.7 min), and *cis*-isorhapontigenin (20.2 min). We presented HPLC-MS-UV chromatographic profiles of the probes with the highest stilbene content (bark and needles, Figure 1a,b). Notably, in the spruce bark extract, *cis*-astringin and *trans*-piceatanol came out as a part of the *trans*-piceid and *trans*-isorhapontin peaks, respectively (Figure 1a), since there were high amounts of *trans*-piceid and *trans*-isorhapontin in these probes. In order to measure these substances, it was necessary to prepare highly diluted extracts of the spruce bark. In other probes with lower levels of stilbenes, the separation of all detected stilbenes was clearer (needles, Figure 1b). *Trans*-astringin, *trans*-piceid, and *trans*-isorhapontin prevailed over other stilbenes reaching 65–99.9% of all detected stilbenoids (Table 1). The most abundant stilbene was *trans*-isorhapontin in the bark (144.3 mg/g DW), which constituted 14.4% of the used bark mass and about 87.5% of all stilbenes in the bark (Table 1).

### 2.2. The Effect of Different Solvents on Stilbene Extraction from the Bark of P. jezoensis

Different solvents, such as ethanol, methanol, or ethyl acetate, were previously applied to optimize stilbene extraction protocol [20,29,30,31]. In this study, we compared the ability of different solvents, including methanol (MeOH), ethanol (EtOH), water (H_2_O), hexane, ethyl acetate, and acetone, to extract stilbenes from the bark of *P. jezoensis*. The data presented in Table 2 demonstrated that extraction of the bark probes with 100% methanol and 96–100% ethanol yielded the highest stilbene levels (152.1–229.1 mg/g DW). Stilbene levels extracted with water, ethyl acetate, and acetone (54.4–87.3 mg/g DW) were 2.4–2.9 times lower than that after methanol or ethanol extraction (Table 2). The lowest level of stilbenes was released using hexane, only 7.7 mg/g DW (Table 2).

### 2.3. The Effect of Different Extraction Temperatures and Extraction Time on Stilbene Yields from the P. jezoensis Bark

Then, we analyzed whether the temperatures of drying the plant material or extraction time could affect stilbene composition and yield. We extracted stilbenes at 20, 40, and 60 °C (temperatures above 60 °C were not used as this could lead to the methanol boiling). Application of lower extraction temperatures (20 and 40 °C) significantly lowered the yield of extracted stilbenes as compared with the incubation at 60 °C (Table 3). Thus, 60 °C was the most suitable temperature for stilbene extraction from the bark of *P. jezoensis*. To analyze whether stilbenes degrade at 60 °C, we increased the extraction time from 2 h to 4 h and 6 h. As shown in Table 3, the content of most stilbenes increased after 4 h and 6 h of incubation compared with the incubation for 2 h at 60 °C, although all these improvements were not statistically significant. The data show that incubation at 60 °C for 4 h did not lead to the destruction of stilbenes. On the contrary, there was a slight increase in stilbene yield, which could be explained by a better extraction efficiency. Additionally, to analyze whether high temperatures could lead to degradation of stilbenes, we heated the commercial standard of *t*-resveratrol at 60 °C for 2 h. The additional data were presented in the supplementary Figure S1 and Table S2. We did not detect any new peaks of substances after a comparison of the HPLC-UV chromatographic profiles for the stilbene standard before and after treatment at 60 °C for 2 h (Figure S1). The amount of the original substance remained approximately at the same level (Table S1). The content of resveratrol only slightly decreased (from 0.901 to 0.898 mg/mL), which is less than 0.4% of the initial amount. The result shows that stilbenes are heat-resistant substances.

### 2.4. Seasonal Variation in Stilbene Content in the Bark of P. jezoensis

It has been shown that stilbene levels in the needles of *P. jezoensis* considerably varied depending on the season [25]. Therefore, we analyzed stilbene repertoire and content in the *P. jezoensis* bark collected at different seasons to assess what time is the most appropriate for sample collection and further stilbene extraction. The bark samples of *P. jezoensis* were collected and extracted in spring, summer, autumn, and winter. The data obtained revealed a considerably higher total content of stilbenes in the winter probes and the lowest—in the summer probes (Table 4). The results confirmed the previously obtained data on seasonal variation of stilbene levels in the needles of *P. jezoensis*, demonstrating that stilbene level was significantly reduced in the *P. jezoensis* material collected in summer [25,26]. The decrease in the total stilbene content in the summer probes was caused by a decrease in the content of two major spruce stilbenes, *t*-astringin and *t*-isorhapontin (Table 4). At the same time, the content of *cis*-isorhapontin sharply increased (up to 6.8 mg/DW) in the summer probes, but this increase was slight in comparison with the decrease in the levels of *t*-astringin and *t*-isorhapontin (Table 4). The data obtained indicate that winter is the most appropriate season for stilbene extraction from the bark of *P. jezoensis*.

## 3. Discussion

Although stilbenes have been detected in plants of more than 30 families, only four plant families were able to accumulate more than 10 mg/g DW of stilbenes [6]. Plant families with the highest total content of stilbenes included Pinaceae (Gymnosperms, Pinidae, conifers), Moraceae (Magnoliopsida, dicotyledons), Polygonaceae (Magnoliopsida, dicotyledons), and Vitaceae (Magnoliopsida, dicotyledons). In other plant species, the total content of stilbenes was considerably lower than 10 mg/g DW or was documented only for certain stilbenes [6]. To the best of our knowledge, the highest content of stilbenes (58–60 mg/g DW) was reported for the roots and stump bark of Norway spruce *Picea abies* and Black pine *Pinus nigra* [27,28], root bark of Mulberry *Morus albus* L. Benxi, Liaoning region 54 mg/g DW [32], roots of Japanese knotweed *Polygonum cuspidatum* 11.1–19.4 mg/g DW [33], and canes of *Vitis vinifera* 10.8–10.9 mg/DW for Vitaceae [34]. Thus, a comparison with stilbene production levels detected for other plant species indicated that the bark of *P. jezoensis* is a rich source of stilbenes. This study showed that the newly analyzed species of spruce *P. jezoensis* resulted in the highest level of total stilbenes (251 mg/g DW) detected in the plant material.

Additionally, we showed that the bark of *P. jezoensis* harvested in winter and spring contained significantly higher levels of stilbenes than the bark collected from summer and autumn spruce. This seasonal variation in stilbene content confirmed previously published results demonstrating that stilbene level was significantly reduced in the *P. jezoensis* material collected in summer [25,35]. It is possible that the seasonal variation in stilbene content contributes to protection of the spruce tissues from adverse environmental conditions, since it is known that stilbene biosynthesis is activated in response to ultraviolet irradiation, drought, salinization, pathogen attack, and other environmental stresses [36]. In addition, the considerable reduction in the content of stilbenes in the bark harvested in summer can be explained by the active growth of the spruce tissues. It is known that cell growth and biomass accumulation compete with the synthesis of secondary metabolites, since these processes employ similar sources of energy and materials for the synthesis of specific compounds. Thus, the detected seasonal variation in the content of stilbenes suggests that spring and autumn could be the most suitable seasons for stilbene extraction from the spruce material; therefore, the data could have an important commercial significance. Thus, the present study firstly reported on the *P. jezoensis* bark as a rich source of stilbenes with stilbene levels exceeding stilbene production detected for other plant species. In addition, this study defined the most effective approaches for stilbene extraction from *P. jezoensis*.

The bark of *P. jezoensis* is a rich source of primarily *t*-isorhapontin (84–87% of total stilbenes in extract) and *t*-astringin (9–12% of total stilbenes in extract). *T*-isorhapontin is a glucoside of isorhapontigenin, which is a tetrahydroxylated stilbenoid with a methoxy group. Currently, little is known about the biological properties of isorhapontin, astringin, and other spruce stilbenes. Several studies reported on the antimicrobial effects of the bark-associated spruce stilbenes on food pathogens and spoilage organisms [37,38], plant fungal pathogens [39], wood-degradative microorganisms [40], and even termites [41].

In conclusion, this study revealed that extract of *P. jezoensis* bark, which is a major waste product in wood industries, is a rich source of stilbenes and could potentially be used as a natural antimicrobial instrument against pathogenic microorganisms. Active studies of medicinal and plant-protecting properties of spruce stilbenes are necessary for their application in biotechnology.

## 4. Materials and Methods

### 4.1. Plant Material

Bark, wood, and needles of of *Picea jezoensis* were harvested from the spruce trunks in the Far Eastern Federal District of Russia. The bark, wood, and needles of *P. jezoensis* were supplied by the Ivanovka sawmill (Russia, longitude 43.97641661616043 and latitude 132.4808241135788) from debarking of logs. The bark was milled and sieved to select particles between 0.2 and 1 mm. The needles, bark, and wood were collected in spring, summer, autumn, and winter of 2019. In the laboratory, the raw material was separated from wood residues, washed, and then dried at 60 °C for 1 day.

### 4.2. Optimization of Stilbene Extraction

Various organic solvents can be used for stilbene extraction and analysis. Although grapevine cane extracts are usually obtained using methanol or ethanol, there are also reports on using other solvents [29,30,42]. To select the most optimal solvent for stilbene extraction from the *P. jezoensis* bark, we used 100 mg of dry mass of crushed and dried bark collected in the winter and extracted stilbenes with methanol (100% and 70%), ethanol (96%, 70%, 40%), water, hexane, ethyl acetate, and acetone at 60 °C for 2 h. Determination of the most optimal temperature and time for stilbene extraction from the dried and crushed bark was carried out using methanol as a solvent at 20, 40, and 60 °C for 2, 4, and 6 h. After extraction, the extract was purified with Discovery^®^ DSC-18 SPE Tube bed wt. 50 mg, volume 1 mL (Supelco, Bellefonte, PA, USA) and then used for the HPLC-MS-UV analysis. Measurements for each sample were repeated 2 times.

### 4.3. High-Performance Liquid Chromatography and Mass-Spectrometry

The identification of individual stilbenes was achieved by a comparison with commercially available standards and HPLC with MS detection. The targeted HPLC with high-resolution mass spectrometry of the stilbene derivatives was carried out using the 1260 Infinity LC analytical system (Agilent Technologies, Santa Clara, CA, USA) equipped with a G1315D photodiode array detector and coupled to an ion trap mass spectrometer (Bruker HCT ultra PTM Discovery System, Bruker Daltonik GmbH, Bremen, Germany) as described [25]. All studied samples were analysed for quantitative determination by HPLC with diode array detection (HPLC-DAD) using an HPLC LC-20 AD XR analytical system (Shimadzu, Japan) as described [43]. All determined components of the crude extracts were identified using the chromatographic and MS data (see Table S2) and were compared with their respective standards. The content of stilbenes was determined by the external standard method using calibration curves of five-point regression, built using the available standards.

The chromatographic separation was performed on a Shim-pack GIST C18 column (150 mm, 2.1 nm i.d., 3-µm part size, Shimadzu, Japan); the column temperature was 40 °C. The mobile phase consisted of A (0.1% aqueous acetic acid) and B (0.1% acetic acid in acetonitrile), which was maintained at a constant flow rate of 0.2 mL/min. The gradient program was used as follows: 0 min 0% of B; 35 min 40% of B; 40 min 50% of B; 50 min 100% of B; and then eluent B until 65 min. The injected volume was 1 µL.

Analytical standards included *t*-piceid obtained from Sigma-Aldrich (St. Louis, MO, USA), *t*-piceatannol obtained from Enzo Life Sciences (New York, NY, USA), *t*-astringin and *t*-isorhapontin obtained from Polyphenols (Sandnes, Norway), and *t*-resveratrol from TCI (Tokyo Chemical Industry UK Ltd., Oxford, United Kingdom). *Cis* forms of stilbenes are not commercially available due to their instability in solid form. For determination of the *cis* isomers of stilbenes, the corresponding standards were obtained in accordance with the method described earlier with minor modifications [25,44,45]. Briefly, 1 mL of the corresponding *trans*-standard (1 mg/mL) was exposed to ultraviolet B (312 nm) at room temperature for 2 h. The concentration of *cis* isomer was calculated according to the difference between the concentrations before and after exposure to UV-B [21].

### 4.4. Statistical Analysis

The data are presented as mean ± standard error and were tested by Student’s *t*-test. The 0.05 level was selected as the point of minimal statistical significance in all analyses. The data on stilbene content were obtained from three different plants with two replicates each.

## Figures and Tables

**Table 1:** Stilbene content and repertoire in the bark, wood, and needles of *Picea jezoensis* (mg per g of the dry weight (DW)) collected in winter. The data were obtained from a representative HPLC profile for the 96% ethanol extracts (60 °C, 2 h) recorded at 310 nm. Means followed by the same letter (a, b, c, or ab) in one row were not different using Student’s *t*-test (three independent experiments). *p* < 0.05 was considered statistically significant.

Stilbenes	Bark, mg/g DW	Needles, mg/g DW	Wood, mg/g DW
*trans*-astringin	16.32 ± 2.20 ^a^	6.49 ± 1.01 ^b^	0.02 ± 0.01 ^c^
*cis*-astringin	0.02 ± 0.01 ^a^	0.02 ± 0.01 ^a^	0.01 ± 0.01 ^a^
*trans*-piceid	4.14 ± 0.30 ^a^	1.36 ± 0.62 ^b^	0.01 ± 0.01 ^c^
*trans*-isorhapontin	144.28 ± 11.38 ^a^	0 ^b^	0 ^b^
*trans*-piceatannol	0.01 ± 0.01 ^b^	0.04 ± 0.01 ^a^	0.02 ± 0.01 ^ab^
*cis*-piceid	0.01 ± 0.01 ^a^	0.02 ± 0.01 ^a^	0 ^a^
*cis*-isorhapontin	0.32 ± 0.09 ^a^	0 ^b^	0 ^b^
*trans*-resveratrol	0.14 ± 0.01 ^a^	0.24 ± 0.07 ^a^	0.01 ± 0.01 ^b^
*trans*-isorhapontigenin	0.02 ± 0.01 ^a^	0.01 ± 0.01 ^ab^	0 ^b^
*cis*-resveratrol	0.02 ± 0.01 ^a^	0.01 ± 0.01 ^ab^	0 ^b^
*cis*-isorhapontigenin	0.01 ± 0.01 ^a^	0 ^a^	0 ^a^
Total	165.29 ± 14.49 ^a^	8.19 ± 1.71 ^b^	0.07 ± 0.02 ^c^

**Table 2:** Stilbene content in the bark of *Picea jezoensis* collected in winter after extraction with different solvents for 2 h at 60 °C (mg per g of the dry weight (mg/g DW)). Means followed by the same letter (a, b, c, d, e, f, g, h, i, ab, bc, or cd) in one row were not different using Student’s *t*-test (three independent experiments). *p* < 0.05 was considered statistically significant.

Stilbenes, mg/g DW	MeOH (100%)	MeOH (70%)	EtOH (96%)	EtOH (70%)	H_2_O	Hexane	Ethyl Acetate	Acetone
*t*-astringin	26.46 ± 2.75 ^a^	20.78 ± 1.88 ^ab^	16.72 ± 0.94 ^b^	16.17 ± 1.43 ^bc^	7.11 ± 0.56 ^e^	0.87 ± 0.22 ^f^	5.91 ± 0.55 ^e^	10.09 ± 0.74 ^d^
*cis*-astringin	0.71 ± 0.22 ^a^	0.59 ± 0.18 ^ab^	0.14 ± 0.06 ^c^	0.17 ± 0.08 ^c^	0.38 ± 0.10 ^b^	0 ^d^	0 ^d^	0 ^d^
*t*-piceid	6.73 ± 0.78 ^a^	5.95 ± 0.79 ^ab^	4.91 ± 0.67 ^b^	4.08 ± 0.59 ^b^	2.14 ± 0.33 ^c^	0.21 ± 0.06 ^e^	1.47 ± 0.12 ^d^	2.40 ± 0.32 ^c^
*t*-isorhapontin	193.16 ± 2.21 ^a^	174.46 ± 1.98 ^b^	148.5 ± 1.77 ^c^	130.79 ± 5.04 ^d^	64.71 ± 2.89 ^g^	6.62 ± 1.16 ^i^	46.80 ± 1.32 ^h^	74.52 ± 1.16 ^f^
*t*-piceatannol	0 ^b^	0.01 ± 0.01 ^b^	0.01 ± 0.01 ^b^	0.01 ± 0.01 ^b^	0.27 ± 0.05 ^a^	0 ^b^	0.01 ± 0.01 ^b^	0 ^b^
*cis*-piceid	0.09 ± 0.04 ^b^	0.12 ± 0.05 ^ab^	0.02 ± 0.01 ^c^	0.06 ± 0.02 ^b^	0.26 ± 0.08 ^a^	0 ^c^	0.01 ± 0.01 ^c^	0 ^c^
*cis*-isorhapontin	1.58 ± 0.11 ^a^	1.59 ± 0.21 ^a^	0.41 ± 0.09 ^c^	0.59 ± 0.09 ^c^	1.04 ± 0.10 ^b^	0 ^e^	0.13 ± 0.07 ^d^	0.27 ± 0.08 ^cd^
*t*-resveratrol	0.16 ± 0.04 ^b^	0.16 ± 0.03 ^b^	0.16 ± 0.05 ^b^	0.20 ± 0.06 ^ab^	0.42 ± 0.12 ^a^	0.01 ± 0.01 ^c^	0.02 ± 0.01 ^c^	0.03 ± 0.02 ^c^
*t*-isorhapontigenin	0.13 ± 0.03 ^a^	0 ^b^	0.02 ± 0.01 ^b^	0.03 ± 0.02 ^b^	0.03 ± 0.02 ^b^	0 ^b^	0 ^b^	0 ^b^
*cis*-resveratrol	0.05 ± 0.03 ^a^	0.05 ± 0.02 ^a^	0.04 ± 0.02 ^ab^	0.02 ± 0.01 ^ab^	0.02 ± 0.01 ^ab^	0 ^b^	0.01 ± 0.01 ^ab^	0.01 ± 0.01 ^ab^
*cis*-isorhapontigenin	0.01 ± 0.01 ^a^	0.01 ± 0.01 ^a^	0.01 ± 0.01 ^a^	0 ^a^	0 ^a^	0 ^a^	0 ^a^	0 ^a^
Total	229.08 ± 6.57 ^a^	203.72 ± 4.12 ^b^	170.94 ± 6.68 ^c^	152.12 ± 7.54 ^c^	76.38 ± 3.70 ^f^	7.71 ± 0.92 ^h^	54.36 ± 2.44 ^g^	87.32 ± 2.87 ^e^

**Table 3:** Stilbene content in the bark of *Picea jezoensis* (mg per g of the dry weight (mg/g DW)) after using different extraction temperatures and times for stilbene analysis. Means followed by the same letter (a, b, c, d, ab, bc) in one row were not different using Student’s *t*-test (three independent experiments). Stilbenes were extracted using methanol from the bark of *P. jezoensis* harvested in winter. *p* < 0.05 was considered statistically significant.

Stilbenes, mg/g DW	2 h, 20 °C	2 h, 40 °C	2 h, 60 °C	4h, 20 °C	4 h, 40 °C	4 h, 60 °C	6 h, 20 °C	6 h, 40 °C	6 h, 60 °C
*t*-astringin	22.92 ± 1.20 ^b^	24.79 ± 0.69 ^ab^	27.46 ± 0.57 ^a^	25.54 ± 1.51 ^ab^	25.93 ± 0.59 ^ab^	28.76 ± 2.07 ^a^	24.94 ± 2.43 ^ab^	25.21 ± 0.96 ^ab^	25.54 ± 3.04 ^ab^
*cis*-astringin	0.61 ± 0.03 ^a^	0.60 ± 0.03 ^a^	0.71 ± 0.05 ^a^	0.64 ± 0.06 ^a^	0.65 ± 0.03 ^a^	0.69 ± 0.04 ^a^	0.68 ± 0.05 ^a^	0.67 ± 0.06 ^a^	0.73 ± 0.08 ^a^
*t*-piceid	5.87 ± 0.31 ^b^	6.09 ± 0.11 ^ab^	6.73 ± 0.32 ^ab^	6.48 ± 0.34 ^ab^	6.49 ± 0.07 ^ab^	7.14 ± 0.26 ^a^	6.13 ± 0.35 ^ab^	6.30 ± 0.18 ^ab^	6.59 ± 0.63 ^ab^
*t*-isorhapontin	175.78 ± 2.64 ^c^	183.31 ± 3.72 ^bc^	204.16 ± 1.27 ^ab^	196.53 ± 4.95 ^b^	199.35 ± 2.38 ^b^	204.14 ± 2.97 ^ab^	207.6 ± 3.92 ^b^	217.06 ± 3.57 ^ab^	214.78 ± 2.75 ^a^
*t*-piceatannol	0 ^a^	0.01 ± 0.01 ^a^	0.01 ± 0.01 ^a^	0.01 ± 0.01 ^a^	0.01 ± 0.01 ^a^	0.01 ± 0.01 ^a^	0.01 ± 0.01 ^a^	0.01 ± 0.01 ^a^	0.01 ± 0.01 ^a^
*cis*-piceid	0.04 ± 0.02 ^b^	0.07 ± 0.01 ^ab^	0.09 ± 0.01 ^a^	0.06 ± 0.02 ^ab^	0.08 ± 0.01 ^ab^	0.09 ± 0.02 ^a^	0.06 ± 0.01 ^ab^	0.08 ± 0.01 ^ab^	0.09 ± 0.01 ^a^
*cis*-isorhapontin	0.82 ± 0.07 ^b^	1.06 ± 0.14 ^b^	1.58 ± 0.11 ^ab^	0.91 ± 0.35 ^b^	1.33 ± 0.58 ^ab^	1.56 ± 0.12 ^ab^	0.91 ± 0.10 ^b^	1.4 ± 0.07 ^ab^	1.79 ± 0.25 ^a^
*t*-resveratrol	0.08 ± 0.02 ^b^	0.09 ± 0.01 ^b^	0.16 ± 0.02 ^a^	0.09 ± 0.04 ^b^	0.12 ± 0.01 ^ab^	0.15 ± 0.04 ^a^	0.10 ± 0.01 ^b^	0.13 ± 0.01 ^a^	0.17 ± 0.02 ^a^
*t*-isorhapontigenin	0.10 ± 0.03 ^ab^	0.08 ± 0.01 ^b^	0.13 ± 0.02 ^a^	0.08 ± 0.01 ^b^	0.10 ± 0.02 ^ab^	0.12 ± 0.02 ^a^	0.08 ± 0.02 ^ab^	0.09 ± 0.02 ^ab^	0.11 ± 0.02 ^ab^
*cis*-resveratrol	0.03 ± 0.01 ^a^	0.03 ± 0.01 ^a^	0.05 ± 0.01 ^a^	0.03 ± 0.01 ^a^	0.03 ± 0.01	0.04 ± 0.01 ^a^	0.03 ± 0.01 ^a^	0.04 ± 0.01 ^a^	0.04 ± 0.01 ^a^
*cis*-isorhapontigenin	0 ^a^	0 ^a^	0.01 ± 0.01 ^a^	0 ^a^	0 ^a^	0.01 ± 0.01 ^a^	0 ^a^	0 ^a^	0.01 ± 0.01 ^a^
Total	206.23 ± 4.14 ^d^	216.15 ± 3.99 ^c^	241.09 ± 3.22 ^ab^	230.38 ± 6.33 ^bc^	234.09 ± 3.94 ^b^	242.71 ± 5.29 ^ab^	240.54 ± 6.63 ^b^	250.99 ± 3.41 ^ab^	249.87 ± 4.85 ^a^

**Table 4:** Seasonal variations in stilbene content and repertoire in the bark of *Picea jezoensis* (mg per g of the dry weight (mg/g DW)). Stilbenes were extracted using ethanol for 2 h at 60 °C. Means followed by the same letter (a, b, c, d, ab, bc, or cd) in one row were not different using Student’s *t*-test (three independent experiments). *p* < 0.05 was considered statistically significant.

Stilbenes	Collected in Spring	Collected in Summer	Collected in Autumn	Collected in Winter
*t*-astringin	16.12 ± 1.74 ^b^	12.65 ± 1.19 ^c^	20.58 ± 1.12 ^a^	23.8 ± 0.69 ^a^
*cis*-astringin	0.54 ± 0.17 ^a^	0.16 ± 0.07 ^b^	0.32 ± 0.09 ^ab^	0 ^c^
*t*-piceid	2.73 ± 0.74 ^b^	3.67 ± 0.36 ^b^	4.28 ± 0.19 ^ab^	4.87 ± 0.12 ^a^
*t*-isorhapontin	130.9 ± 18.37 ^ab^	104.35 ± 11.7 ^b^	112.98 ± 5.13 ^b^	134.26 ± 5.54 ^a^
*t*-piceatannol	0.19 ± 0.08 ^a^	0.02 ± 0.02 ^b^	0.05 ± 0.01 ^b^	0 ^c^
*cis*-piceid	0.04 ± 0.03 ^b^	0.03 ± 0.01 ^b^	0.21 ± 0.02 ^a^	0 ^c^
*cis*-isorhapontin	0.19 ± 0.08 ^b^	6.8 ± 3.3 ^a^	0 ^c^	0 ^c^
*t*-resveratrol	0.18 ± 0.05 ^cd^	0.32 ± 0.09 ^bc^	0.56 ± 0.05 ^a^	0.07 ± 0.04 ^d^
*t*-isorhapontigenin	0.04 ± 0.03 ^ab^	0.22 ± 0.08 ^a^	0 ^b^	0 ^b^
*cis*-resveratrol	0 ^a^	0 ^a^	0.01 ± 0.01 ^a^	0.01 ± 0.01 ^a^
*cis*-isorhapontigenin	0.01 ± 0.01 ^ab^	0.03 ± 0.01 ^a^	0 ^b^	0 ^b^
Total	150.95 ± 19.93 ^ab^	126.54 ± 15.06 ^b^	138.97 ± 6.42 ^b^	163.02 ± 6.36 ^a^

## Data Availability

Data are contained within the article and Supplementary Materials.

## Supplementary Materials

The following are available online at https://www.mdpi.com/article/10.3390/metabo11110714/s1, Supplemental Figure S1: Comparison of HPLC-UV chromatographic profiles for the stilbene standard before (a) and after treatment at 60 °C for 2 h recorded at 310 nm, Table S1: List of the stilbene derivatives identified in the extracts of *Picea jezoensis* bark, Table S2: Resveratrol content before and after treatment by 60 °C during 2 h.
