# Supplementary material for: The Bark of the Spruce Picea jezoensis Is a Rich Source of Stilbenes
Source: Metabolites. 2021 Oct 20;11(11):714. doi: 10.3390/metabo11110714 (PMC8625855; doi:10.3390/metabo11110714)
Supplement: Supplementary file 1 [file metabolites-11-00714-s001.zip › metabolites-1426101-supplementary.pdf]

Figure S1. Comparison of HPLC-UV chromatographic profiles for the stilbene standard before (A) and after (B) treatment at 60ºC for 2h recorded at 310 nm.

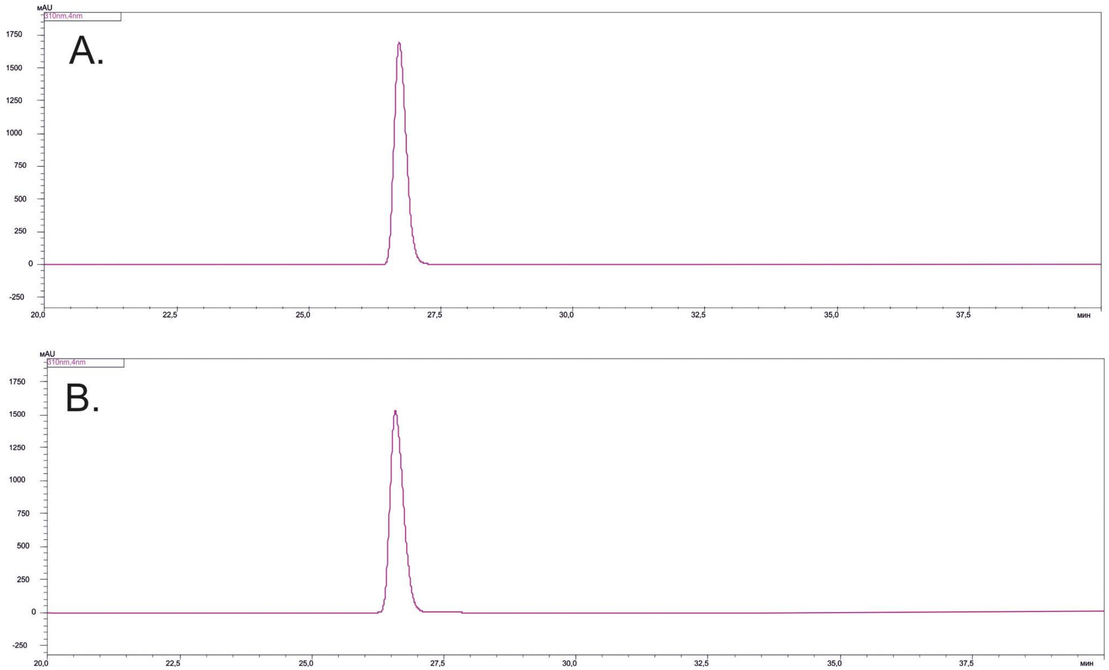

Supplemental Table S1. List of the stilbene derivatives identified in the extracts of *Picea jezoensis* bark.

Peak no.	Rt (min)	Negative ion mode			Elemental composition	MS^2 fragmentation (precursor ions $[M–H]^–$) (*m/z*)	UV, λmax (nm)	Assignment
		$[M–H]^–$ (*m/z*)	$[2M–H]^–$ (*m/z*)	$[M–H+AcOH]^–$ (*m/z*)				
1	18.0	405.1	811.2	465.1	$C_{20}H_{22}O_9$	243, 225, 201	324	*trans* -astringin

2	20.2	405.1	811.2	-	$C_{20}H_{22}O_9$	243, 225	291	*cis*-astringin
3	20.8	389.1	779.3	449.1	$C_{20}H_{22}O_8$	227	317	*trans* -piceid
4	21.9	419.1	839.2	479.2	$C_{21}H_{24}O_9$	257, 242, 241	326	*trans* -isorhapontin
5	22.6	243.0	487.1	303.1	$C_{14}H_{12}O_4$	225, 215, 201, 200, 199, 185, 175, 173, 159, 157	324	*trans* -piceatannol
6	23.9	389.1	779.2	449.1	$C_{20}H_{22}O_8$	227	285	*cis*-piceid
7	24.5	419.1	839.2	479.2	$C_{21}H_{24}O_9$	257, 242, 241	283	*cis*-isorhapontin
8	26.5	227.1	455.2	287.2	$C_{14}H_{12}O_3$	185, 227	308	*trans*-resveratrol
9	28.8	257.1	514.1	317.1	$C_{15}H_{14}O_4$	224, 242, 257	324	*trans*-isorhapontigenin
10	29.7	227.1	455.2	287.2	$C_{14}H_{12}O_3$	185, 227	290	*cis*-resveratrol
11	30.2	257	514.1	317.1	$C_{15}H_{14}O_4$	224, 242, 257	285	*cis*-isorhapontigenin

Supplemental Table S2. Resveratrol content before and after treatment by 60ºC during 2h.

Compound	Resveratrol concentration before treatment by 60ºC during 2h, mg/ml	Resveratrol concentration after treatment by 60ºC during 2h, mg/ml
Resveratrol	0.901	0.898
